# Supplementary material for: Introns of plant pri-miRNAs enhance miRNA biogenesis
Source: EMBO Rep. 2013 May 17;14(7):622–8. doi: 10.1038/embor.2013.62 (PMC3701235; doi:10.1038/embor.2013.62)
Supplement: Supplementary Information [file embor201362s1.pdf]

Figure S1

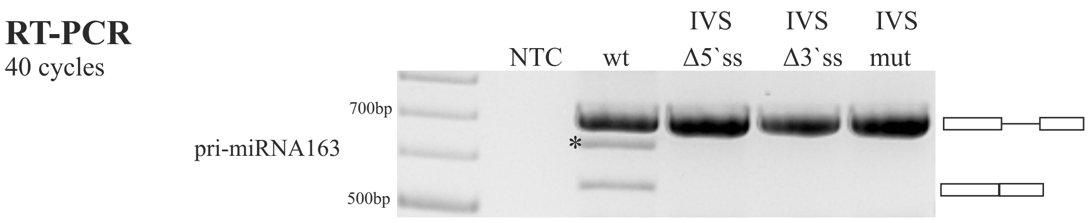

Fig. S1 Transcripts generated from the *MIR163* gene variants in which splice sites have been mutated are not spliced.
RT-PCR products were separated on a agarose gel stained with ethidium bromide.
wt: wild type *MIR163;* IVSΔ5`ss: *MIR163* with the 5`ss mutated; IVSΔ3`ss: *MIR163* with the 3`ss mutated; IVSmut: *MIR163* with both splice sites mutated. NTC stands for non template control; * marks an unidentified DNA fragment.

Figure S2

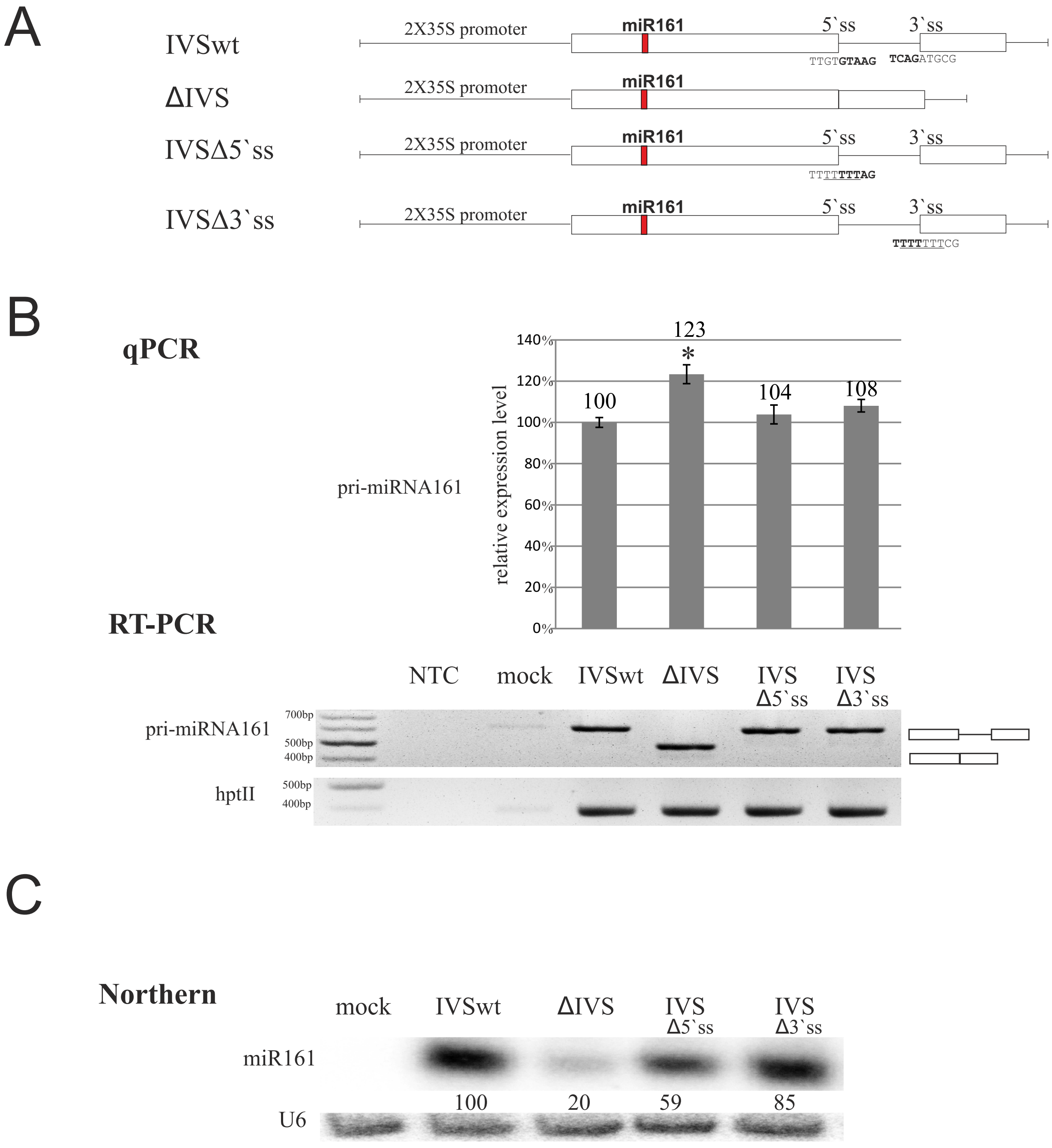

D

RT-PCR
40 cycles

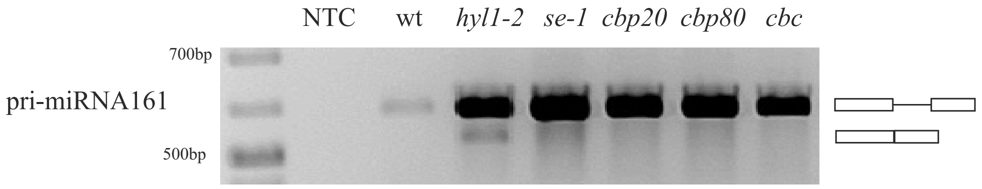

Fig. S2 The intron of *MIR161* stimulates the biogenesis of miR161. The proper accumulation of miR161 requires the functional 5'ss.
(A) Schematic representation of *MIR161* gene variants used. The splice sites and position of miRNA (marked in red) are shown.
(B) Level of pri-miR161 by real-time PCR (qPCR, upper panel) and RT-PCR (lower panel) recorded in infiltrated *Nicotiana benthamiana* leaves expressing the *MIR161* variants shown in (A). The expression of the hygromycin phosphotransferase gene (hptII) serves as a positive control of agroinfiltration. Error bars indicate SD (n=3), and an asterisk indicates a significant difference between the indicated and IVSwt sample (Mann-Whitney test, $p<0.05$).
(C) Level of miR161 recorded by Northern blot hybridization using RNA samples isolated from inflitrated leaves expressing the *MIR161* variants shown in (A). U6 serves as a loading control. Levels of miRNA expression in mutant plants are compared to those observed in wt plants marked as 100%.
(D) Level of pri-miR161 recorded by semiquantitative RT-PCR in wild type Arabidopsis plants and miRNA biogenesis mutants (*hyl1-2, se-1, cbp20, cbp80* and *cbc* which is the *cbp20cbp80* double mutant). The spliced pri-miRNA cannot be detected in wt plants since it is rapidly processed. This explains why the spliced version of pri-miR161 is not observed in *N. benthamiana* infiltration experiments shown in Fig. S2B.
NTC stands for a non template control. Mock is a negative control obtained by infiltration of *N. benthamiana* leaves with MES buffer.

Figure S3

Northern

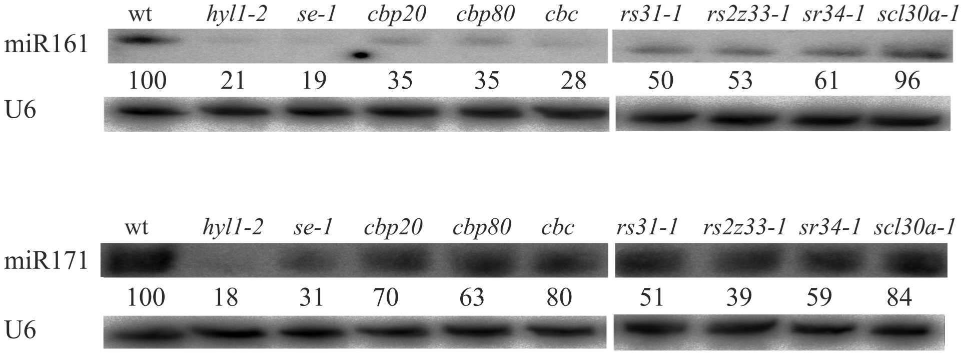

Fig. S3 SR proteins affect biogenesis of miR161 and miR171.
The levels of miR161 and miR171were detected by Northern blot hybridization in wt Arabidopsis plants as well as in selected miRNA biogenesis mutants (*hyl1-2, se-1, cbp20, cbp80* and *cbc* which is the *cbp20cbp80* double mutant) and SR protein mutants (*rs31-1, rs2z33-1, sr34-1, scl30a-1*). U6 serves as a loading control. Levels of miRNA expression in mutant plants are compared to those observed in wt plants marked as 100%.

Figure S4

pri-miR163 – both polyA isoforms

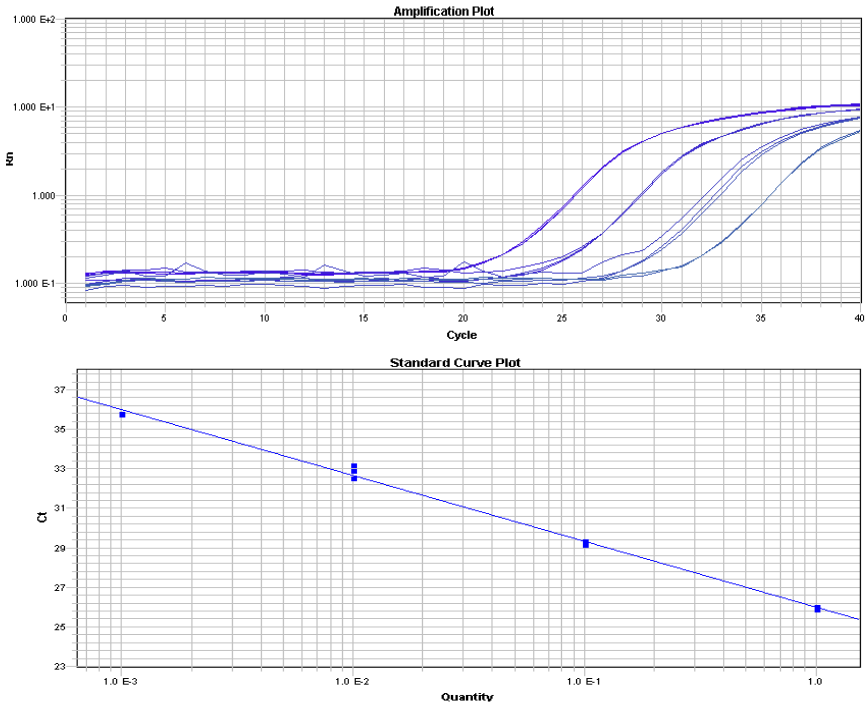

Slope: -3.3287
Efficiency: 0.99
R^2: 0.996

pri-miR163 – isoform with the distal polyA

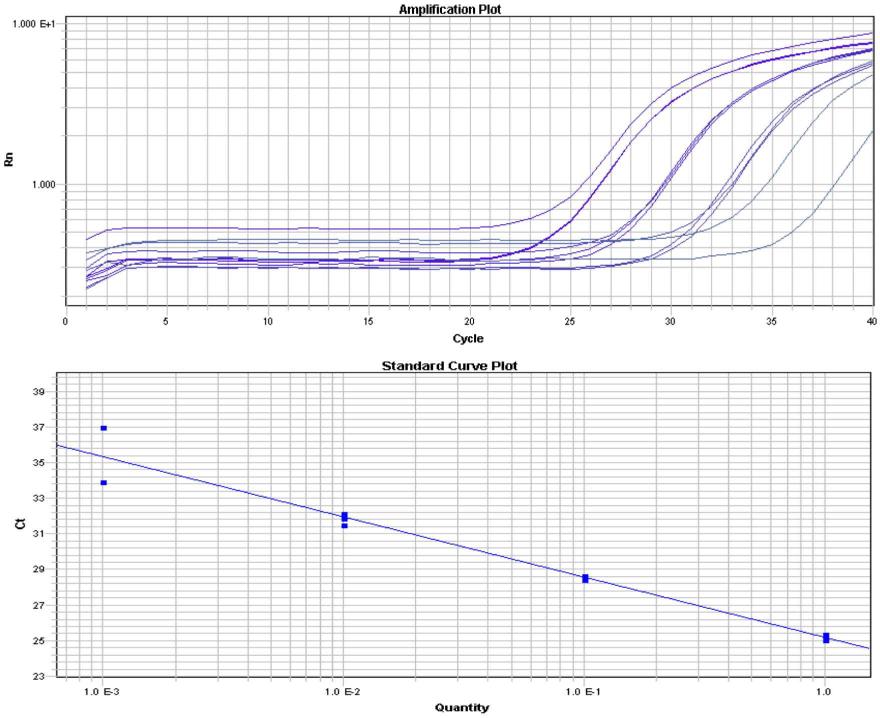

Slope: -3.383
Efficiency: 0.98
R^2: 0.965

Fig. S4. Evaluation of real-time PCR reactions designed to determine the relative abundance of two alternatively polyadenylated isoforms of pri-miR163. Serial cDNA dilutions were used as templates to determine the efficiencies of both PCR reactions. Calibration curves show that the efficiencies are very similar, thus allowing a direct comparison and estimation the levels of polyadenylated isoform abundance.

Table S1 Oligonuleotides used in this study

Primers used for constructs preparation

Name	Sequence	cDNA/gene fragment amplified using the primer pair	Constructs prepared using the amplified fragment
A01	TTGCGGCCGCTCGTGAATCTTTGTTTCCTC	promoter + *MIR163*	IVSwt (Fig. 1A)
A02	TTGGCGCGCCCAAGCGTCCAGACTTCAG		
A03	TTGCGGCCGCTCGTGAATCTTTGTTTCCT	promoter + 1st exon of *MIR163*	ΔIVS (Fig. 1A)
A04	GAGGAAACAAAAAATTTCCGTTATCTCTTTTCATC		
A05	GATGAAAAGAGATAACGGAAATTTTTTGTTTCCTC	2nd exon of *MIR163*	
A06	TTGGCGCGCCCAAGCGTCCAGACTTCAG		
A07	GATGAAAAGAGATTTTTTTAGTCATGCACATG	mutagenesis of IVSwt *MIR163*	IVSΔ5`ss; IVSmut (Fig. 2A)
A08	CATGTGCATGACTAAAAAAATCTCTTTTCATC		
A09	GTCTAATGATTTTTTTTTAATTTTTTGTTTC	mutagenesis of IVSwt *MIR163*	IVSΔ3`ss; IVSmut (Fig. 2A)
A10	GAAACAAAAAATTAAAAAAAAATCATTAGAC		

Name	Sequence	cDNA/gene fragment amplified using the primer pair	Constructs prepared using the amplified fragment (Fig. S2 A)
A11	TTGCGGCCGCCACTTATCTCTAACTCATCC	*MIR161*	IVSwt
A12	TTGGCGCGCCTGTCTTCTTCTTCTCTTGTG		
A13	TTGCGGCCGCCACTTATCTCTAACTCATCC	1st exon of *MIR161*	ΔIVS
A14	CTTTTAAAAACTTTCTCGCATCACAATTTCAATGCTTTTCC		
A15	GGAAAAGCATTGAAATTGTGATGCGAGAAAGTTTTTAAAAG	2nd exon of *MIR161*	
A16	TTGGCGCGCCTGTCTTCTTCTTCTCTTGTG		
A17	CATTGAAATTTTTTTTGGAGATGGATATG	mutagenesis of IVSwt *MIR161*	IVSΔ5`ss
A18	CATATCCATCTCCAAAAAAAATTTCAATG		
A19	GTTCAATTGTTATTTTTTTTTTTCGAGAAAGTTTTTAAAAG	mutagenesis of IVSwt *MIR161*	IVSΔ3`ss
A20	CTTTTAAAAACTTTCTCGAAAAAAAAAAATAACAATTGAAC		

Primers used for RT-PCR, qPCR and 5`RACE analyses

Name	Sequence	cDNA/gene fragment amplified using the primer pair	Experiments in which the primer pair was used
B01	GAGAGTGAGAAAAATAAAGAG	pri-miR163	RT-PCR (Fig.1-3)
B02	AGGATGTTGACACGTGTAAAC		
B03	AGTACCTTAGATAAACCGACCAAAACC	pri-miR163 (1st exon)	qPCR; poly(A) ratio (Fig. 2C)
B04	AACCGGGAACTCCAGCACTT		
B05	GAATGCAAATGGTTGTGGAA	pri-miR163 (2nd exon)	
B06	GGGCCACTAAAGCCCTTAAA		
B07	GGCATCAGATTTCACCTTTTTC	pri-miR161	qPCR (Fig. S2 B)
B08	CAAATGATGCAATCTCAAACAAA		
B09	ATTTCGGCTCCAACAATGTC	HPTII	qPCR (Fig. S2 B)
B10	GATGTTGGCGACCTCGTATT		

B11	TGCCGAAGCTTTGATCAGTA	pri-miR161	RT-PCR (Fig. S2 B, D)
B12	TCAAATGATGCAATCTCAAACA		
B13	ACATTGTTGGAGCCGAAATC	HPTII	RT-PCR (Fig. S2 B)
B14	GTGCTTGACATTGGGGAGTT		
B15	GGGATCTCGGACATCTTAACTCCGTCTCTC	At1g66690	5` RACE (Fig. 4A)
B16	CCCCGGAGGGAGTGTCTGGA	At1g66690	qPCR (Fig. 4B)
B17	GGCGATCTCCGCCAACCTCG		

Primers used as probes in Northern blot hybridizations

**Name**	**Sequence**	**Detection of miRNA**
C01	ATCGAAGTTCCAAGTCCTCTTCAA	miR163 (Fig.1-4)
C02	CGTGATATTGGCACGGCTCAA	miR171 (Fig. S3)
C03	TAGTCACTTTCAATGCATTGA	miR161 (Fig. S3)
